# Experiences and challenges of implementing clinical medication reviews in daily practice: a mixed-methods study

**DOI:** 10.1007/s11096-025-01992-2

**Published:** 2025-09-08

**Authors:** Sek Hung Chau, Petra J. M. Elders, Jacintha Domić, H. J. Marjorie G. Nelissen-Vrancken, François G. Schellevis, Jacqueline G. Hugtenburg

**Affiliations:** 1 Department of Clinical Pharmacology and Pharmacy, Amsterdam UMC Location Vrije Universiteit Amsterdam, De Boelelaan 1117, Amsterdam, The Netherlands; 2 Amsterdam Public Health, Quality of Care, Amsterdam, The Netherlands; 3 Department of General Practice, Amsterdam UMC Location University of Amsterdam, Meibergdreef 9, Amsterdam, The Netherlands; 4 Dutch Institute for Rational Use of Medicine, Utrecht, The Netherlands; 5 NIVEL (Netherlands Institute for Health Services Research), Utrecht, The Netherlands

**Keywords:** Aged, Community pharmacy services, General practitioners, Medication review, Patient participation, Pharmacist, Pharmacy technicians

## Abstract

**Introduction:**

Organisational problems still prevent widespread implementation of clinical medication reviews. The Opti-Med2 method was developed to facilitate the process of performing clinical medication reviews. The method includes patient involvement by means of a questionnaire and expert teams of community pharmacists and general practitioners (GPs) to perform pharmacotherapeutic analyses, providing the patients’ own GP with pharmacotherapeutic advice. There is a supporting role of community pharmacy technicians and general practice nurses/assistants in the process.

**Aim:**

To gain insight into the implementation of the Opti-Med2 method within the framework of pharmacotherapeutic audit meeting groups in the Netherlands.

**Method:**

A mixed-methods implementation study in seven groups of primary care healthcare providers. Quantitative data were collected using study forms. Semi-structured interviews with 8 GPs, 5 community pharmacists and 2 community pharmacy technicians were held. Interviews were transcribed verbatim and were analysed using the extended Normalization Process Theory.

**Results:**

Only one group provided sufficient quantitative data for analysis. Of the pharmacotherapeutic advice given by the expert team, 72% was adopted by the GPs of which 85% resulted in an intervention with the patient. In general, the healthcare providers were satisfied with using the Opti-Med2 method. The use of expert teams was appreciated by most GPs and community pharmacists. All healthcare providers were very satisfied with the use of the patient questionnaire.

**Conclusion:**

Although full implementation of Opti-Med2 method as a whole was not achieved, the structured organisation of conducting CMRs and the use of questionnaires was deemed successful.

**Supplementary Information:**

The online version contains supplementary material available at 10.1007/s11096-025-01992-2.

## Impact statements

- The Opti-Med2 method increases the efficiency of the clinical medication review process, thereby allowing healthcare providers to conduct a larger annual number of clinical medication reviews in daily practice.
- The availability of a patient questionnaire to support the anamnesis of the patient in clinical medication reviews enables easier delegation of tasks to pharmacy staff, for example to take the pharmacotherapeutic anamnesis.
- Using the Opti-Med2 method considerably enhances patient input and their involvement in the clinical medication review process.

## Introduction

The Dutch 2012 multidisciplinary guideline ‘Polypharmacy in the Elderly’ contributed to the development of enhanced patient-oriented pharmacotherapeutic (PT) care for older people with chronic conditions [1]. An important element of this care is the periodic evaluation of the medication prescribed to this vulnerable patient group by conducting clinical medication reviews (CMRs) also referred to as PCNE type 3 medication reviews [2]. Importantly, a CMR also includes examining how patients experience the use of their medication and what their personal treatment goals are [1, 3]. Explicit involvement of patients in the CMR process facilitates the identification and management of drug-related problems (DRPs), and reduces the occurrence of new DRPs [4]. Since 2015, the Dutch Health Care Inspectorate therefore requires community pharmacies and general practitioners (GP) practices to conduct at least 100 and 20 CMRs annually, respectively [5].

Based on research and practical experience, it has been recommended to conduct CMRs only in patients with a high risk of DRPs and use a systematic approach on the basis of collaborative agreements between community pharmacists (CPs) and GPs [6, 7]. Although progress has been made, some persistent bottlenecks, in particular the absence of a clear structured method/procedure for conducting CMRs and the time it takes to prepare and implement a CMR, still prevent a broad and successful implementation in daily practice [6, 8]. It is therefore highly important to enhance the efficiency of the CMR process. Central to this is making the process as simple as possible while maintaining a systematic approach and using the available knowledge and resources through existing interprofessional partnerships as efficiently as possible. Delegating tasks to staff members could contribute to the efficiency of conducting CMRs and has also been addressed in studies in the US [9–11], Canada [12], Germany [13–15] and the Netherlands [16].

Considering the above, the Opti-Med method for conducting a CMR was developed in a previous study [6, 17, 18]. Details of the Opti-Med method are provided in Table 1.

**Table 1:** The elements of the Opti-Med method and adaptations in Opti-Med2

	Opti-Med method	Adaptations in Opti-Med2
1	The use of a questionnaire to be completed by the patient as a replacement for the anamnesis	The use of a questionnaire to be completed by the patient as a replacement or preparation for the anamnesis, if necessary with the help of a practice nurse or community pharmacist technician
2	File composition (diagnoses, laboratory values and medication overview) by the practice nurse in collaboration with a community pharmacist technician	
3	PT analysis using a digital medication review tool and drafting a proposal for an PT treatment plan by an external independent expert team including a GP or elderly care physician and a community pharmacist not involved instead of the patients’ own GP and CP	A digital medication review tool is not provided, however, the GP and CP use their own medication review tool if available The expert team consists of a fixed pair of a GP and a CP within the Pharmacotherapy Audit Meeting Group
4	Feedback to and discussion of the PT treatment plan with the patient by the own GP	Feedback to and discussion of the PT treatment plan with the patient by the own GP or the CP
5	Follow-up and monitoring	

Interviews with participating healthcare providers (HCPs) revealed that this method led to a meaningful and pleasant collaboration between HCPs. The complementary knowledge of CPs and GPs/elderly care physicians in the expert teams increased their willingness to jointly take critical decisions on pharmacotherapy, while most patients rated their CMR as (very) helpful [19]. The promising experiences with Opti-Med provided the basis for the implementation of CMRs.

**Fig. 1:**
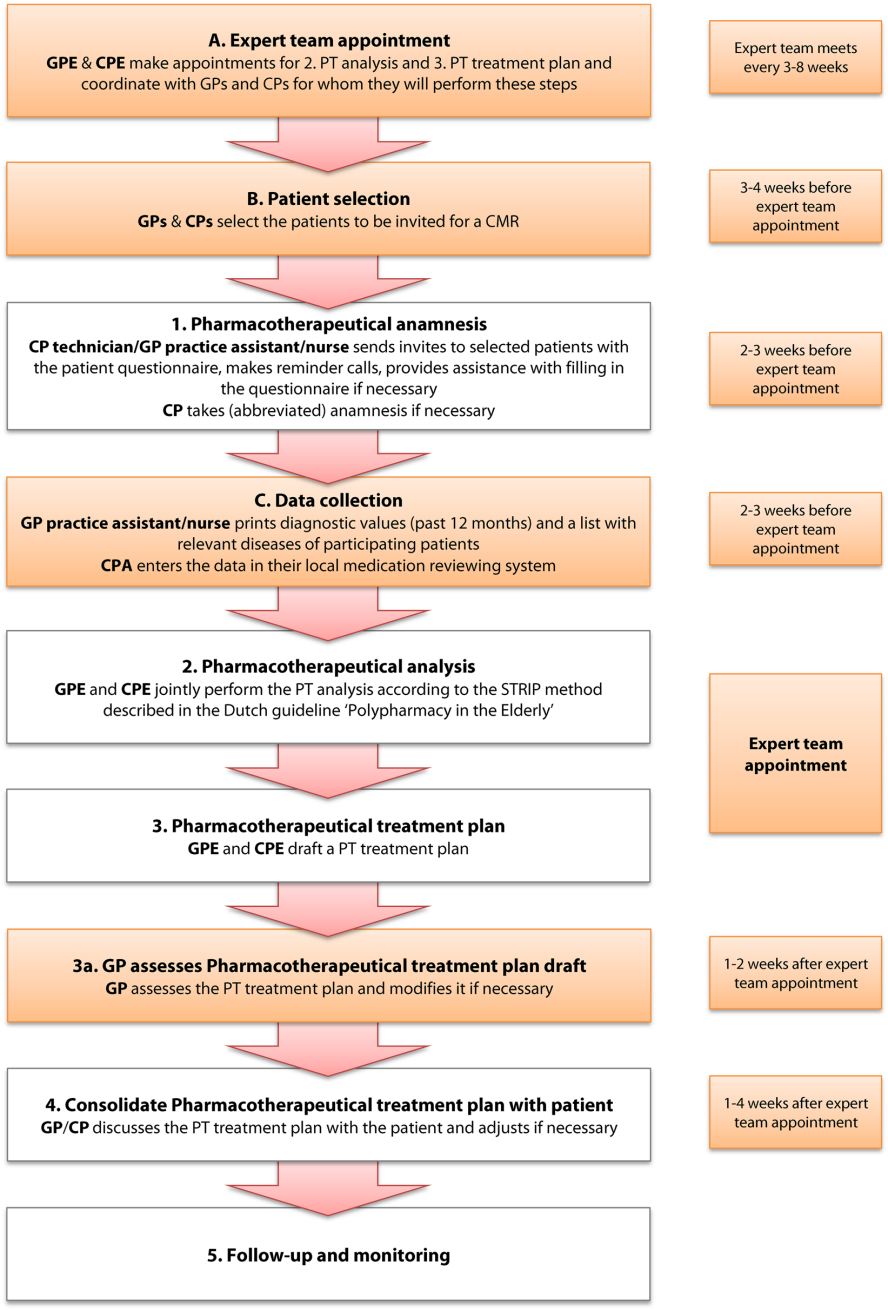
The Dutch multidisciplinary guideline ‘Polypharmacy in the Elderly’ divides the approach of a CMR in five steps, labelled 1 to 5 (clear rectangles). The supporting steps introduced with Opti-Med2 are labelled from A to C (orange rectangles). GPE = General Practitioner Expert team member, CPE = Community Pharmacist Expert team member, PT = Pharmacotherapeutic, GP = General Practitioner, CP = Community Pharmacist, CMR = clinical medication review, STRIP = Systematic Tool to Reduce Inappropriate Prescribing

Some adjustments had to be made, as the external expert team was funded by the trial and the digital medication review tool used is not publicly available (Fig. 1). From the Opti-Med elements, the formation of expert teams including a GP and a CP, the questionnaire to obtain the patient’s perspective and the deployment of GP practice nurses/assistants and/or community pharmacy assistants (CPAs) were implemented in the present study.

More than 90% of Dutch CPs and GPs attend pharmacotherapeutic audit meetings (PTAMs). PTAMs have evolved into an interprofessional education and collaboration activity to improve PT policy by making agreements about the prescribing of medication at the population level [20, 21]. The PTAM group therefore seemed an obvious platform for implementing CMRs using Opti-Med.

### Aim

The aim of the study was to gain insight into the implementation of the Opti-Med method (Opti-Med2) within the local PTAM framework and whether it could be implemented in clinical care.

### Ethics approval

The Medical Ethics Review Committee of VU University Medical Center deemed on 25/01/2018 that the Dutch Medical Research Involving Human Subjects Act did not apply to this study (FWA00017598), and ethical approval was therefore not required. Expert team participants provided written informed consent to participate and interview participants provided consent to record the interview.

## Method

### Study design

A mixed methods study of the implementation of Opti-Med2 involving primarily PTAM groups in the period January 2019–December 2020. Both quantitative data and interviews were used.

### Participants

PTAM groups in various regions were recruited to participate in an implementation study through existing contacts with care groups, the newsletter of the Institute for Rational Medicine Use (IVM) and calls on the websites of the Royal Dutch Association for the Advancement of Pharmacy (KNMP) and the Dutch College of General Practitioners (NHG). During recruitment, initiatives to conduct CMRs had already started. Therefore, the inclusion criterion to recruit only PTAM groups that have at least five annual meetings and which make concrete agreements was replaced by the requirement that at least one expert team member had already followed formal training to conduct CMRs.

### Implementation

Three meetings were held with the members of each local expert team with the aim to familiarising them with Opti-Med2 and supporting the implementation (Table 2) [22].

**Table 2:** Implementation meetings

Kick-off meeting	The researcher (SC) discussed and explained Opti-Med2, the optional elements and workbook [22]. After exchanging views, agreements were made on the selection criteria of patients to review (age, living situation, use of multi-dose dispensing system among others), the number to review, which modules of Opti-Med2 they would use, the distribution and delegation of tasks, how to select and invite the patients and how to exchange patient information (after patient consent). The researcher would provide each group a flowchart after the meeting that summarised the local agreements and as an aid to visualise the process. (A general shortened questionnaire is included in Supplementary material A.)
Mid-term evaluation	An appointment was planned a few weeks after the kick-off meeting to evaluate the process and progress and to identify if the process needs adjustment. A topic list was used (see Supplementary material B1)
Final evaluation	After approximately 12 weeks, a semi-structured interview was conducted, aimed to understand the barriers and facilitators of the implementation of conducting CMRs using Opti-Med2. For the topic lists, see Supplementary material B2 and B3

*CMRs* Clinical medication reviews

### Outcomes

1. Experiences of HCPs with Opti-Med2 when conducting CMRs.
2. Barriers and facilitators for the implementation of Opti-Med2.

### Quantitative measurements

Participating groups were requested to record several details of the CMRs conducted: the PT advice given by the expert team, whether the PT advice was implemented or not, time spent on the PT analysis and CMR process respectively on a registration form (Supplementary Material C).

### Interviews

Semi-structured face-to-face and telephone interviews were conducted with a convenience sample of CPs, GPs, CPAs, and GP practice nurses/assistants by SC, JH, JD and BvdW. Views and experiences regarding the implementation of the Opti-Med2 method were explored using the four constructs of the extended Normalization Process Theory (eNPT): potential, capacity, capability and contribution. The topics of the interview guide included: motivation, roles, materials, resources, workability, knowledge and skills, expectations, experiences and reflections regarding Opti-Med2. A variety of professions in different roles (expert team member or not) and varying working and geographical environments (within/outside health centre, degree of urbanisation, socioeconomic status scores of the population [SES-WOA]) to include a variety of perspectives on PT care were included. The degree of urbanisation, municipal size and SES-WOA were derived from 2019 Statistics Netherlands data [23–25].

### Data analysis

The interviews were audio-recorded and transcribed in a naturalised manner. The transcripts were independently analysed by three researchers (SC & PE or JH) using the eNPT as a theoretical framework [26]. The coding was discussed until consensus was reached; should controversy remain, a third researcher (PE or JH) was consulted to reach a decision.

eNPT includes of four constructs. The first three constructs consist of the social-cognitive resources that are available to HCPs, i.e. *potential*, possibilities that are offered by Opti-Med2, i.e. *capability*, and social-structural resources available to HCPs, i.e. *capacity*. The final construct indicates what HCPs do to implement Opti-Med2, i.e. *contribution* [26].

Interview data were managed with MaxQDA 2020. Descriptive quantitative analyses were performed using IBM SPSS Statistics for Windows, Version 26.0.

## Results

### Participants

Members of 40 PTAM groups, either pharmacists (35) or GPs (5) were asked to participate. Seven groups agreed to participate. Characteristics are shown in Table D1 (Supplementary Material D).

One group did not start because the GPs were ultimately not willing to change the process of conducting CMRs. One group withdrew after implementing the questionnaire and task delegation to pharmacy technicians after the CP indicated it was too much effort, and in one group the CP expert left the pharmacy halfway through the project. Four groups were ongoing with Opti-Med2 until the end of the study. Although participating groups were asked to record details about the CMRs conducted, only sufficient data from one group was received. Reasons for not providing data were: forgotten to register; forms were lost; considering recording data an additional task; data only being recorded digitally in the GP information system, but not on the study forms.

### CMRs performed

Six participating groups conducted on mean 28 ± 31 CMRs during their participation. One PTAM reported to have conducted 22 CMRs with 54 proposed interventions, of which 39 (72.2%) were implemented.

### Interviews

#### Characteristics of interviewees

Fifteen HCPs were interviewed separately at their workplace. Their characteristics are shown in Table 2. The interviews lasted 10 to 34 min with an average of 21 min.

#### Potential

##### Individual intentions

Most CPs and GPs stated that conducting CMRs is important. A CPA and a GP mentioned that CMRs could improve the quality of the PT treatment of patients. Patient involvement was considered as an important quality asset. The HCPs also believed that the contact between GPs and CPs went more smoothly as the result of an intensified level of collaboration. Showing the patient that CPs and GPs actively collaborate was seen as positive and promoting patient adherence was also mentioned as a positive point.

Nearly all HCPs recognised that participating in the study was an opportunity to improve the CMR process. An advantage mentioned by many HCPs was the gain in efficiency, which allowed for an increase in the number of CMRs conducted. Both GPs and CPs saw a possibility to achieve continuity in the conductance of CMRs. Several CPs hoped that their appointments with GPs to perform PT analyses would be cancelled less often and that they would be able to better embed CMRs in their daily activities. A GP was motivated to try a new approach because of dissatisfaction with the current outcomes of CMRs.

*“And we already do, let’s say, medication reviews. And, every year we end up with the same people with the same things (…) so maybe it would be interesting to try this once, to see if we can change things this way.” – General Practitioner Expert, group 7, participant 1 (GPE7.1).*

##### Shared commitment

Jointly conducting the project was mentioned by HCPs as a success factor. In one group, the intervention did not materialise because too few GPs appeared willing to participate.

*“However, the doctors want to keep it separate for each doctor. They don't want one doctor to discuss the reviews for the other doctors.” – Community Pharmacist Expert, group 1, participant 1 (CPE1.1).*

#### Capacity

##### Social norms

A pharmacy technician reported that most patients were open to discussing their medication or to complete a questionnaire, but that some patients only wanted to discuss these matters with their doctor. On the other hand, a GP mentioned that patients spontaneously remarked that they appreciated being contacted and counselled by the CP.

*“Yes, I thought that was excellent. So the pharmacy starts, and I notice from the patients that they also like it very much.” – General Practitioner non Expert, group 2, participant 4 (GPnE2.4)*

##### Social roles

Several GPs and CPs had reservations about other HCPs reviewing their patients. A CP mentioned that it might be confusing for the patient and another CP was concerned about potential competition between the pharmacies. A GP felt somewhat uncomfortable about giving feedback to his colleagues, while another GP and a CP expected that the approach in which other HCPs were involved in PT analyses could lead to new insights.

*“Of course, you’re a little bit under a magnifying glass. If your colleague assesses it, and you get an advice about it, yes. Yes, it has to be safe for that, I think, but I dare to do that.” – GPE7.1*

Some CPs appreciated the contribution of pharmacy technicians and GP practice nurses in conducting CMRs. Pharmacy technicians were enthusiastic about these extended responsibilities (Table 3). A pharmacy technician mentioned she was able to deepen the relationship with the patient.

**Table 3:** Characteristics of interviewed healthcare professionals

#	Respondent	Profession	Role	Gender
1	CPE1.1	Community pharmacist	Expert	Female
2	GPE1.1	Community pharmacist	Expert	Female
3	GPE2.1	General practitioner	Expert	Female
4	CPE2.2	Community pharmacist	Expert	Male
5	CPE2.3	Community pharmacist	Expert	Female
6	GPnE2.4	General practitioner	Non expert	Female
7	CPT2.5	Community pharmacy technician	–	Female
8	CPT2.6	Community pharmacy technician	–	Female
9	CPE4.1	Community pharmacist	Expert	Female
10	GPE4.2	General practitioner in training	Expert	Female
11	CPE5.1	Community pharmacist	Expert	Female
12	GPE5.2	General practitioner	Expert	Male
13	GPE5.3	General practitioner	Expert	Female
14	GPnE5.4	General practitioner	Non expert	Male
15	GPE7.1	General practitioner	Expert	Male

*CPE* Community Pharmacist Expert team member; *GPE* General Practitioner Expert team member; *GPnE* General Practitioner, not in expert team, *CPT* Community pharmacy technician

A CP was concerned that by having patients reviewed by other HCPs, the individual relationship with the patient as well as with the other HCP could be harmed. Another fear was that the centralisation of CMRs could give healthcare insurers an incentive to impose centralisation on a larger scale. This could lead to bypassing local HCPs and more-table top reviews which are cheaper but certainly much less patient-oriented.

*“If one pharmacist for the whole city starts reviewing then, as far as I'm concerned it's really theoretical paper reviews and, is not patient-centred.” – CPE2.3*

##### Material resources (instruction manual, invitation letter, questionnaire)

All groups used the patient questionnaire. A CP sent the questionnaire but additionally invited patients for a face-to-face interview. Other CPs and pharmacy technicians only invited patients when they were unable to fill in the questionnaire themselves. Some CPs and pharmacy technicians remarked that the questionnaire was used as a discussion aid during the anamnesis with the patient and helped to time manage the interview. A GP considered some questions too difficult for older people.

*“Yes, the questions have become more difficult for many people. Most of them are over the 80s. (..) My advice would be (..), fill that in with their child. – GPE7.1*

The invitation letter inviting patients to complete the questionnaire was deemed useful. Patients appreciated that it was signed by both GP and CP, which made clear that it was a joint effort.

#### Capability

##### Workability

Several HCPs remarked they had to find a practical way to deal with the questionnaire. Most GPs, CPs and pharmacy technicians were satisfied with using a questionnaire. Some mentioned that conveying the expert teams’ PT advice to the patients’ GP took additional time and led to impoverishment of information. GPs who received PT advice, mentioned that it took little effort to implement the advice.

*“I can only write it down very briefly for a colleague. If you've been there, then you know exactly what it's about and then you also know the reason why something might be changed.” – GPE2.1*

HCPs were satisfied with the expert team. CPs appreciated that they had one contact for multiple practices, which simplified the organisation of CMRs. A GP feared that the organisation would be problematic and difficult to fit into the busy practices of GPs, but it all went smoothly.

*“We really did ten each session. And then GPE5.2 also ten, approximately. So that's twenty patients per month, so that's a lot more than we expected.” – GPE5.3*

##### Integration

GPs and CPs mentioned that having fixed appointments was crucial to keeping the review process going. A CP described that in case an appointment was cancelled, it was difficult to get back on track. Another CP described that collaboration came to a standstill after an expert team member went on leave.

*"Well, particularly in that, we have really, now, started planning it. So every time we meet, we plan a new date." – GPE5.3*

A GP said that a simple procedure was introduced by placing the PT advices in their physical mailbox, which made implementing PT advices a part of routine.

#### Contribution

##### Coherence

Not knowing the patient was mentioned as an obstacle by many HCPs, because patient records often lacked information required for conducting CMRs. GPs receiving PT advices reported that interventions were not always appropriate.

Other HCPs mentioned it was easier to look at the medication more objectively and critically when a patient is unknown.

*“And that did make it a bit easier to look at the matter from a distance, actually. And then fewer emotions come into play, because I don't know all those people. So then you come to very clear conclusions.” – GPE7.1*

GPs experienced that by performing more PT analyses, they became more efficient because they had learned from previous sessions. A GP mentioned that GPs interested in CMRs and who conduct them regularly, can develop expertise which is otherwise difficult to develop.

Most GPs noticed changes in medication after a CMR. Some were deemed important, such as stopping medicines, others as minor.

*“We observed things that had gone very wrong which I had not expected. Indeed, medicines that should have been stopped a long time ago and things like that.” – GPE5.3*

##### Cognitive participation

GPs and CPs noted that the intensive collaboration during the project improved interprofessional collaboration. A CP remarked that the project also increased job satisfaction. Another CP was happy about the more frequent appointments for the analyses. A CP also noted that GPs increasingly apply the skills and knowledge acquired during CMRs in daily practice.

A GP appreciated the additional value of the perspective of the CP in the quality of the CMRs. A CP noted the importance of mutual respect for each other’s profession and expertise.

##### Collective action

Each group took their own approach to setting up the expert group. In most groups, GPs reviewed their own patients as well as those of their colleagues, whereas the CPs only reviewed their own patients. In one group, a GP only reviewed patients from his colleagues in the GP group practice.

Several GPs and CPs made clear agreements on performing patient selection. Some delegated administrative tasks to pharmacy technicians or GP practice assistants.

A GP remarked that they had adopted and implemented PT advices for interventions in most cases. It was mentioned that some recommendations were not followed up by the GP and were noticed to be open-ended after several months. This seemed more common when GPs were not involved in an expert team. Sometimes this is caused by the high time demands of daily practice. In other occasions, interventions proposed by the expert team in the medical record of the patient's own GP could be missed, because newer entries covered the earlier ones. This problem was circumvented by adding a pop-up appearing on the GP's screen when accessing the patient’s file.

*"But especially those things that then also need to be discussed with the patient, when further explanation is needed, in the day-to-day, hecticness is not picked up. Then three months later, these questions are still open." – CPE2.3*

##### Reflexive monitoring

Several HCPs reported continuing conducting CMRs using expert teams. HCPs in one group expressed their plans to create multiple expert teams within the group, one per GP practice instead of one for all practices, and maintain the questionnaires, task delegations and appointment structure.

Several groups shortened and/or adapted the questionnaire. Most CPs and pharmacy technicians considered the shortened questionnaire more useful.

*"By a coincidence I used the short version and my colleague in the other pharmacy the long version. It turned out that, that with us the questionnaires were sent in better than with my colleague." – CPE2.2*

One CP considered the questionnaire the most valuable intervention element. Some CPs mentioned to have used the questionnaire for patients reviewed outside the expert team, while the use of the questionnaire as a valuable tool also spread to other partner pharmacies.

##### Barriers and facilitators for the implementation of Opti-Med2

An overview of the barriers and facilitators for the implementation of Opti-Med2 classified according to the eNPT framework is presented in Table 4. Facilitators include: 1. the structured approach based on the PTAM framework allowed for achieving the required number of CMRs; 2. the intensified HCP collaboration contributed to good relationships between CPs and GPs; 3. pharmacy staff members expressed enthusiasm about their newly-assigned tasks and 4. the use of the Opti-Med2 method reduced the time investment to conduct CMRs.

**Table 4:** Barriers and facilitators according to the eNPT-framework

eNPT construct	Barriers	Facilitators
Potential		Healthcare providers already had experience in conducting CMRs Pharmacists have received training to be able to conduct CMRs Pharmacist technicians communicate with patients on a daily basis Improving adherence The number of CMRs conducted increased DRPs were quickly identified and resolved Stop unnecessary medications
Capacity	Not wanting another doctor to do the PT analysis of the patients	Structured approach based on the PTAM facilitates the organisation of CMR Intensive cooperation contributes to the good relationship between GP and pharmacist Pharmacist technicians are excited about their new duties
Capability	Searching patient files that the GP does not know takes a lot of time Failure to find the treatment advice of the expert team in the patient file by the own GP. This was solved by a pop-up in the GP screen	Reduced time investment through: Using the questionnaire, Pharmacist technicians contribute to data collection and organisation, Structured consultation within the expert team leads to efficient PT analysis, Discussion of the treatment plan with the patient by the own GP
Contribution	The initial questionnaire was too long and was therefore modified Some questions in the questionnaire are not clear or appropriate in their current form The questionnaire is less suitable for people with impaired cognition	The use of the questionnaire will continue

*eNPT* Extended Normalization Process Theory, *CMR* Clinical medication review, *PTAM* pharmacotherapeutic audit meeting, *PT* Pharmacotherapeutic, *DRP* Drug-related problem

Main barriers include: 1. Not wanting any other GP than their own GP to perform the PT analysis, 2. Searching files of patients who the GP does not know is time-consuming. 3. The questionnaire is less suitable for people with impaired cognition.

## Discussion

This study explored issues influencing the implementation of the Opti-Med2 method in daily practice. The full protocol of this project, with a core element of an expert team, was not implemented in most groups. However, nearly all groups retained Opti-Med2 elements in their CMR procedures. The use of the patient questionnaire was adopted in all groups and was considered highly useful. Participating CPs were found more likely to delegate CMR tasks than GPs. Experiences with reviewing medications of patients of other GPs varied. All HCPs indicated that they had the intention to continue using one or more components of the Opti-Med2 method after the study.

### Efficiency of Opti-Med2

Although Opti-Med2 as a tool to improve and speed-up the CMR process was generally well-received, this study revealed that some elements require further adjustment. At the start of the study, it quickly became clear that the patient questionnaire that was initially used was too long. After evaluation, simplification and shortening, the revised questionnaire proved to be highly satisfactory in practice. This questionnaire, however, proved still less suitable for use by people with impaired cognition. This almost inevitable problem can be solved by involving informal caregivers or by going through the questionnaire orally with the patient. The availability of the questionnaire also made it possible to involve pharmacy technicians in the preparation of the PT analysis and to interview patients by telephone or video. These options were used most widely during the COVID-19 pandemic and the subsequent years.

### Staff involvement

Another Opti-Med2 element was the delegation of tasks to pharmacy technicians and GP practice assistants. Some CPs delegate tasks, ranging from administrative tasks to taking anamnesis with the patient. The latter was enabled by deploying the questionnaire as a discussion aid. Pharmacy technicians who were given a role were enthusiastic and saw it as an opportunity to provide PT care. Delegating tasks in the community pharmacy has been studied in the US [9–11] and reported and suggested in Canada [12], Germany [13–15] and the Netherlands [16] and showed that delegation could improve efficiency and enable CPs to provide better care and reach more patients.

### Perspectives of GPs

Reviewing medication of patients registered with other GPs was met with mixed success by the GPs. The implementation did not materialise in one PTAM group because the associated GPs preferred to review their own patients, whereas in another group they planned to form multiple expert teams for each GP practice. In line with the initial Opti-Med trial [27], some GPs considered a limited amount of PT advices as less relevant, but not as bothersome. Also in line with the outcomes of this study was the finding that HCPs recognized the ability to review medication more objectively as an important advantage and would like to review more or even only unknown patients.

As such, the introduction of expert teams allowed GPs to develop expertise by performing a higher number of PT analyses and improved both CMR efficiency and HCP collaboration. This is in line with HCP views expressed in the Opti-Med trial [27].

### Participating in Opti-Med2

Recruiting proved to be difficult. Most PTAM groups that declined participation indicated that they had already started their own approach, which is not surprising, as the Netherlands is at the forefront of conducting CMRs [27]. Most Dutch HCPs feel trained and competent in conducting CMRs [28], in contrast to HCPs in other countries [9, 14, 29]. After relaxing the PTAM inclusion criteria, inclusion improved.

### Implementation of Opti-Med2

Successful implementation of a new working method is influenced by several factors related to the design of the innovative intervention, such as the complexity of the intervention and the procedures needed to correctly implement it, and to the competences of the people involved in implementation such as the level of knowledge required for a successful implementation and the willingness to participate [30]. In addition, the setting and circumstances under which users have to perform the intervention also determine its success. In this case, it concerned preconditions including the need to effectively conduct the intervention, the site of action (the PTAM or the individual pharmacies and GP practices) and the availability of appropriate financial compensation as well as sufficient time. Finally, socio-political factors can also play a role. The most obvious of these is the obligation imposed by the government and health insurers to conduct a minimum number of CMRs annually. The extent to which these factors are reflected in the implementation process not only determines the initial success of the intervention, but also whether the change is sustainable [30]. In each (PTAM) group, these factors differentially influenced the Opti-Med2 implementation process and its outcome.

### Strengths and limitations

Participants practicing within various working and geographical environments were recruited, which contributed to the representativeness of this study.

Although the project required closer collaboration between HCPs than the PTAM structure was intended for, it provided a useful platform to deepen HCP interaction and thereby improve the CMR process.

Most participants recorded less quantitative data than was planned. Time constraints have also been found a common barrier in other pharmacotherapeutic care studies [31, 32].

Participants may have experienced recall bias while reflecting on the use of the Opti-Med2 method, which may have reduced the reliability of the findings. It is also not known whether data saturation has been reached. In addition, because only a few participants from less successful groups could be interviewed, the experiences of these groups may be underrepresented. It is not known what worked for them or did not work for them and why they declined to participate in an interview. Nevertheless, by performing a process evaluation on the basis of the evidence-based methodology of the eNPT [26], valuable insight was gained into the extent to which the method or parts thereof can be used routinely in daily practice.

## Conclusion

Although the Opti-Med2 method was not fully implemented, the structured approach of conducting CMRs and the use of questionnaires proved to be a success. Application of the method is especially useful in settings in which it is difficult to conduct CMRs. In conducting CMRs, there is also a need for digital, multidisciplinary collaborative solutions to support this shared care.

## Supplementary Information

Below is the link to the electronic supplementary material.

Supplementary file A (DOCX 36 kb)

Supplementary file B (DOCX 29 kb)

Supplementary file C (DOCX 27 kb)

Supplementary file D (DOCX 28 kb)
