# Supplementary material for: Experiences and challenges of implementing clinical medication reviews in daily practice: a mixed-methods study
Source: Int J Clin Pharm. 2025 Sep 8;48(2):435–45. doi: 10.1007/s11096-025-01992-2 (PMC12992465; doi:10.1007/s11096-025-01992-2)
Supplement: Supplementary file 1 — Supplementary file A (DOCX 36 kb) [file 11096_2025_1992_MOESM1_ESM.docx]

| **Questionnaire** |
| --- |

| Name: | m/f | Date of birth: |  |
| --- | --- | --- | --- |

I give permission for my GP and my pharmacist to exchange information and consult to conduct a medication review.

|  |
| --- |

Signature:

A doctor other than your own GP **may** review whether changes to your medication are necessary. This doctor also has medical confidentiality, using your current data in your GP's file. A proposal for any changes will first go to your own GP and he/she will assess the proposal before discussing it with you.

🞏 I have no objection to another doctor assessing my medication.

🞏 I do object to another doctor assessing my medication.

| **I. General questions** |  |
| --- | --- |

**1.** How many times have you **fallen** in the past six months?

🞏 Not fallen 🞏 1 time

🞏 2 times or more 🞏 I don't know

**2.** Are you **afraid** of **falling**?

🞏 No 🞏 Yes

| **3.** What is **your height** in centimetres? |  | cm |
| --- | --- | --- |

| **4.** What is your **weight** in kilograms? |  | kg |
| --- | --- | --- |

| **II. Medication questions** |
| --- |

1. **What medicines are you currently taking?**

Would you please write down on page 4 which medicines you are currently taking?

1. **Do you ever use medicines or food supplements that you buy at the drugstore, for instance?** For example, paracetamol, ibuprofen, vitamins, homeopathic remedies or cough syrups?

🞏 No 🞏 Yes → If so, which ones? And how often do you use them?

|  |
| --- |
|  |
|  |
|  |
|  |

1. **What do you expect from your medicines?**

|  |
| --- |
|  |
|  |

1. **In the past month, have you experienced any particular side effects from your medicines?**

🞏 No 🞏 Yes → If so, from which side effects?

| **Side effect**  **(type of complaint)** | **By which medicine?**  If you do not know, fill in a ‘?’. | **How long have you been suffering from this?** |
| --- | --- | --- |
| 1 |  |  |
| 2 |  |  |
| 3 |  |  |

1. **Are you concerned about possible side effects?**

🞏 No 🞏 Yes → If so, about which side effects?

|  |
| --- |
|  |

1. **Are you taking medications that you doubt will work for you?**

🞏 No 🞏 Yes → If so, about which medicine?

|  |
| --- |
|  |

1. **Many people who take several medicines sometimes forget to take a medicine. Have you ever forgotten to take one or more medicines in the past month?**

🞏 No 🞏 Yes → If so, how often? And which medicine was this?

|  |
| --- |
|  |

1. **How do you make sure you don't forget your medicines?**

You may tick multiple boxes

🞏 Use at fixed moments 🞏 Pill box

🞏 Alarm clock, alarm, telephone 🞏 Pre-packed bags per day/medication roll

🞏 Someone helps me 🞏 None of these answers

| 🞏 Otherwise, namely |  |
| --- | --- |

1. **In the past month, have you ever deliberately skipped a medicine or taken less than prescribed?**

🞏 No 🞏 Yes → If so, which medicine was this? And why?

|  |
| --- |
|  |

1. **In the past month, have you ever taken more of one or more medicines than prescribed by the doctor?**

🞏 No 🞏 Yes → If so, which medicine was this? And why?

|  |
| --- |
|  |

1. **Have you stopped taking a particular medicine in the past month without consulting your doctor?**

🞏 No 🞏 Yes → If so, which medicine was this? And why?

|  |
| --- |
|  |

1. **Do you ever struggle to take your medicines as prescribed by the doctor?** You may tick multiple boxes

🞏 No

🞏 Yes, because there are side effects/I am afraid of side effects

🞏 Yes, because I don't feel like it

🞏 Yes, because one or more medicines do not work

| 🞏 Yes, for other reasons, namely |  |
| --- | --- |

1. **Do you ever have practical problems using your medicines?**

🞏 No

🞏 Yes, because I have problems with the times in the day

🞏 Yes, because I find it difficult to swallow the pill/capsule

🞏 Yes, because the medicine strip or package is difficult to open

| 🞏 Yes, for other reasons, namely |  |
| --- | --- |

1. **Are you concerned about anything else?**

|  |
| --- |
|  |
|  |
|  |
|  |

1. **Do you have any questions or comments about your medicines or your health?**

|  |
| --- |
|  |
|  |
|  |
|  |

1. **Would you like to have a conversation about your medicines?**

🞏 No 🞏 Yes, in the pharmacy 🞏 Yes, at home 🞏 Yes, by phone

| **Name medicine** | **Strength** | **How often do you take this per day?** | **What are you using it for?** |
| --- | --- | --- | --- |
|  |  |  |  |
|  |  |  |  |
|  |  |  |  |
|  |  |  |  |
|  |  |  |  |
|  |  |  |  |
|  |  |  |  |
|  |  |  |  |
|  |  |  |  |
|  |  |  |  |
|  |  |  |  |
|  |  |  |  |
|  |  |  |  |
|  |  |  |  |
|  |  |  |  |
