# Supplementary material for: Experiences and challenges of implementing clinical medication reviews in daily practice: a mixed-methods study
Source: Int J Clin Pharm. 2025 Sep 8;48(2):435–45. doi: 10.1007/s11096-025-01992-2 (PMC12992465; doi:10.1007/s11096-025-01992-2)
Supplement: Supplementary file 2 — Supplementary file B (DOCX 29 kb) [file 11096_2025_1992_MOESM2_ESM.docx]

Supplementary Material B – Topic lists evaluations

## Supplementary Material B1 – Topic list Mid-term evaluation

**Exchanging experiences**

What is your experience so far with the Opti-Med method?

- What went well?
- What could have been better?
- How is it going with the organisation?

(time investment & planning)

- Tools used
- Which practical bottlenecks did you encounter?
- Which content-related bottlenecks did you encounter?
- (Do you also manage to perform medication analyses for other doctors?)

**Results**

What are your expectations?

- What goal(s) had you set at the start (explicitly or not)?
  - number of reviews, number of doctors and pharmacists in expert teams, turnaround time
- What have the medication reviews achieved so far?
- How many patients have had their medication reviewed?
- How many medication changes have been suggested by the expert team?
- And how many have been implemented by the GP?
- To what extent have the goals been achieved (and if not, why not)
- Possibly: what were the most common changes?

How did you like it?

Looking back, at what points would you do things differently, why and how?

Do you plan to continue with the project? (Facilitators/barriers)

Supplementary Material B2 – Topic list Final evaluation experts

**Motivation**

- What was your motivation to participate?

**Roles**

- How was the division of roles during the project? Clear?

**Materials (time, money, staff)**

- How did you perform medication reviews before?
- What was the difference of the Opti-Med2 project for you compared to what you did before?
- How much time did you need for
  - the analyses?
  - the treatment plan?
  - discussing it with the patient?
- Can you identify successful/positive elements in the Opti-Med method of working that contributed to high-quality and efficient medication assessments?
  - the structure?
  - preparation of treatment plan?
  - consultation, and consensus or not between doctor and pharmacist?
  - your own knowledge/training?
  - not knowing the patient?
  - the time investment?
- What did you think of the materials offered?
  - questionnaire
  - workbook
  - ...
- Can you list barriers in the Opti-Med method of working that contributed to barriers to good quality and efficient medication reviews or, on the contrary, contributed to less good or less efficient medication reviews?
- What do you think of the materials?

**Knowledge and skills**

- Did you encounter any problems in terms of knowledge or skills when conducting medication reviews?

**Workability**

- How is organisation going (time investment & planning)?
- Tools used
- What practical bottlenecks do you encounter?
- What content-related bottlenecks do you encounter?
- Do you also manage to perform medication analyses for other doctors?

**Revenue**

- What was your expectation for Opti-Med?
- What goal(s) had you set at the start (explicit or not)?
  - number of reviews, number of doctors and pharmacists in expert teams, turnaround time
- What have the medication reviews achieved so far? Are you satisfied or disappointed?

**Reflection**

- Are you satisfied/disappointed?
- Looking back, at what points would you do things differently, why and how?
- What did you think of the support for the project?
  - What was missing/what could be better?
  - What was good/pleasant/useful?

**Future**

- Which point would you approach differently, why and how?
- Do you plan to continue with it? (Facilitators/barriers)

Supplementary Material B3 – Topic list Final evaluation non-experts

Questions in *italics and grey* depending on available time and situation.

**Roles**

- Was the division of roles during the project clear?

**Material (time, money, staff)**

- *How did you perform medication reviews before?*
- *What was the difference of the Opti-Med2 project for you compared to what you did before?*
- How much time did you need for
  - the treatment plan?
  - discussing it with the patient?
- *Can you identify successful/positive elements in the Opti-Med method of working that contributed to high-quality and efficient medication assessments?*
  - *the structure?*
  - *your own knowledge/training?*
  - *the time investment?*
- What did you think of the advice form?
- Can you list barriers in the Opti-Med method of working that contributed to barriers to good quality and efficient medication reviews or, on the contrary, contributed to less good or less efficient medication reviews?
- What do you think of the materials?

**Workability**

- How is organisation going (time investment & planning)?
- What practical bottlenecks do you encounter?
- What content-related bottlenecks do you encounter?

**Results**

- What have the medication reviews yielded for you?
- What have the medication reviews yielded for the patient?

**Reflection**

- Are you satisfied/disappointed?
- Looking back, at what points would you do things differently, why and how?
- What did you think of the support for the project?
  - What was missing/what could be better?
  - What was good/pleasant/useful?

**Future**

- Which point would you approach differently, why and how?
- Do you plan to continue with it? (Facilitators/barriers)
