# Supplementary material for: Experiences and challenges of implementing clinical medication reviews in daily practice: a mixed-methods study
Source: Int J Clin Pharm. 2025 Sep 8;48(2):435–45. doi: 10.1007/s11096-025-01992-2 (PMC12992465; doi:10.1007/s11096-025-01992-2)
Supplement: Supplementary file 3 — Supplementary file C (DOCX 27 kb) [file 11096_2025_1992_MOESM3_ESM.docx]

| To be filled in by expert team | To be filled in by patient’s (own) GP |
| --- | --- |

| **Medication analysis** | | |
| --- | --- | --- |
| Name patient: | Date of birth: | Name GP: |
| Date medication analysis: | Performed by: | |

| **Proposed changes/advice:** | **Explanation/reason:** | **Advice accepted by GP?** | **Agreement patient/ implemented?** |
| --- | --- | --- | --- |
|  |  |  | **Date consult with patient:** |
|  |  | yes/no  explanation: | yes/no  explanation: |
|  |  | yes/no  explanation: | yes/no  explanation: |
|  |  | yes/no  explanation: | yes/no  explanation: |
|  |  | yes/no  explanation: | yes/no  explanation: |
|  |  | yes/no  explanation: | yes/no  explanation: |
|  |  | yes/no  explanation: | yes/no  explanation: |
|  |  | yes/no  explanation: | yes/no  explanation: |
|  |  | yes/no  explanation: | yes/no  explanation: |
|  |  | yes/no  explanation: | yes/no  explanation: |
|  |  | yes/no  explanation: | yes/no  explanation: |
| **Other actions GP:** | | | **Agreement patient/ implemented?** |
|  | | | yes/no  explanation: |
|  | | | yes/no  explanation: |
|  | | | yes/no  explanation: |
| **Please note:**   - **Communicate changes to the pharmacist** - **Put changes on paper for the patient if necessary** | | | |

Continued on reverse side of form -->

| **Time spent** | |
| --- | --- |
| Time medication analysis expert team |  |
| Was the patient’s own GP part of the expert team? | yes/no |
| Time spent preparing for consultation by own GP |  |
| Time spent on patient consult by patient’s own GP |  |
| Time spent after patient consult by patient’s own GP |  |
| Time spent by pharmacist |  |
| Time spent by other healthcare provider (specify) |  |

| **Medication advice** | | | | | | | | | | | |
| --- | --- | --- | --- | --- | --- | --- | --- | --- | --- | --- | --- |
| How useful did you find the medication advice? | | | | | | | | | | | |
| Not useful at all = 1 | 1 | 2 | 3 | 4 | 5 | 6 | 7 | 8 | 9 | 10 | Very useful = 10 |
| Comments/feedback towards expert team | | | | | | | | | | | |
| *Think about: what went well/not well? What could be improved? Do you have any comments?* | | | | | | | | | | | |
| How satisfied was the patient with the medication review process? | | | | | | | | | | | |
| Not satisfied at all = 1 | 1 | 2 | 3 | 4 | 5 | 6 | 7 | 8 | 9 | 10 | Very satisfied = 10 |
| *Explanation* | | | | | | | | | | | |
