# Supplementary material for: Experiences and challenges of implementing clinical medication reviews in daily practice: a mixed-methods study
Source: Int J Clin Pharm. 2025 Sep 8;48(2):435–45. doi: 10.1007/s11096-025-01992-2 (PMC12992465; doi:10.1007/s11096-025-01992-2)
Supplement: Supplementary file 4 — Supplementary file D (DOCX 28 kb) [file 11096_2025_1992_MOESM4_ESM.docx]

Supplementary Material D – Tables supporting the Results section

## Table D1 – Characteristics participants

| Group | Province | Degree of urbanisation and municipal size ^1)^ | SES-WOA ^1)^ | Health centre | Composition expert team | Medication analyses for |
| --- | --- | --- | --- | --- | --- | --- |
| 1 | A | Strongly urbanised, 50.000-100.000 citizens | above average | within | 1 GPE  1 CPE | 2 GPs in 2 GPPr and 1 CPh |
| 2 | B | Strongly urbanised, 150.000-250.000 citizens | below average | within and outside | 1 GPE  3 CPEs | 5 GPs in 3 GPPr and 2 CPh |
| 3 | C | Hardly urbanised,  20.000-50.000 citizens | above average | outside | 1 GPE  1 CPE | 3 GPs in 1 GPPr and 1 CPh |
| 4 | D | Extremely urbanised, 100.000-150.000 citizens | average | outside, also outside PTAM group | 1 GPE  1 CPE | 4 GPs in 3 GPPr and 1 CPh |
| 5 | A | Extremely urbanised,  > 250.000 citizens | average | within | 2 GPEs  1 CPE | 7 GPs in 1 GPPr and 1 CPh |
| 6 | A | Extremely urbanised,  > 250.000 citizens | above average | outside | 1 GPE  1 CPE | 2 GPs in 2 GPPr and 1 CPh |
| 7 | B | Strongly urbanised, 150.000-250.000 citizens | average | outside | 1 GPE  1 CPE | 3 GPs in 1 GPPr and 1 CPh |

Each province was assigned a letter in the alphabet in order of inclusion.

1) According to the 2019 classification of Statistics Netherlands ^24,25^

Abbreviations: SES-WOA = socioeconomic status scores of private households, divided in tertiles; GPE = General Practitioner Expert team member; CPE = Community Pharmacist Expert team member; GP = General Practitioner; GPPr = General Practitioner Practice; CPh = Community Pharmacy; PTAM = pharmacotherapeutic audit meeting.

## Table D2 – Quantitative summary of the CMRs of group 2

| **Characteristics CMRs** |  |
| --- | --- |
| Number of medication analyses with complete follow up (*n*) | 21 |
| Total number of identified DRPs and advice (*n*) | 54 |
| - Median (IQR) | 2 (2-3) |
| Number of proposals accepted/total number of proposals (percentage) | 39/54 (72.2%) |
| Patient’s own GP part of expert team (percentage) | 13 (61.9%) |
| Patient’s own GP not part of expert team (percentage) | 8 (38.1%) |
| **Advice acceptance** |  |
| Advice accepted by GP (*n* = 54) |  |
| Yes | 39 (72.2%) |
| No | 13 (24.1%) |
| Other intervention | 1 (19%) |
| Unknown | 1 (19%) |
| Advice accepted by patient (*n* = 40) |  |
| Yes | 34 (85.0%) |
| No | 3 (7.5%) |
| Not applicable | 2 (5.0%) |
| Unknown | 1 (2.5%) |
| **Satisfaction advice (range 0-10)** |  |
| Grade advice (*n* = 17) | 7.5 |
| - Own GP part of expert team (*n* = 12) | 7.5 |
| - Own GP not part of expert team (*n* = 5) | 7.6 |
| Grade patient (*n* = 14) | 7.6 |
| - Own GP part of expert team (*n* = 9) | 7.7 |
| - Own GP not part of expert team (*n* = 5) | 7.4 |
| **Time spent per component (minutes)** |  |
| - Expert team GPE & CPE (*n* = 19) | 10.3 |
| - Preparation consultation by patient’s own GP (*n* = 18) | 7.8 |
| - Consultation time patient’s GP (*n* = 17) | 8.2 |
| - Time needed after consultation (*n* = 18) | 5.8 |
| - Time CP (*n* = 6) | 10 |
| **Lead time from advice to consultation with patient in days** |  |
| All patients (*n* = 14) |  |
| - Median (IQR) | 8.5 (2-29) |
| - Range | 0-62 |
| Own GP part of expert team (*n* = 11) |  |
| - Median (IQR) | 7 (2-28) |
| - Range | 0-62 |
| Own GP not part of expert team (*n* = 3) |  |
| - Median | 29 |
| - Range | 23-29 |
